# Torsades De Pointes in a 71-Year-Old Female With Normal Qt Interval After Azithromycin Use

**DOI:** 10.7759/cureus.37653

**Published:** 2023-04-16

**Authors:** Allison Foster, Ivan Cancarevic, Muhammad Haseeb ul Rasool, Mahmoud Alashry, Muhammad Ghallab, Nazaakat Ahmed, Sanna Salam, Most Munira

**Affiliations:** 1 Internal Medicine, Icahn School of Medicine at Mount Sinai, Queens Hospital Center, New York City, USA; 2 Medicine, Icahn School of Medicine at Mount Sinai, Queens Hospital Center, New York City, USA; 3 Internal Medicine, Queens Hospital Center, New York City, USA; 4 Medicine, Queens Hospital Center, New York City, USA; 5 Cardilogy, Icahn School of Medicine at Mount Sinai, Queens Hospital Center, New York City, USA

**Keywords:** arrhythmia, cardiomyopathy, qt interval prolongation, azithromycin, torsades de pointes

## Abstract

A 71-year-old female visiting from Colombia presented to the emergency room with a productive cough, subjective fever, and chills for the past three days. Baseline EKG demonstrated a QT interval of 385 milliseconds with left ventricular hypertrophy and T wave inversions in leads V4, V5, and V6. Azithromycin was administered, and she was subsequently found to have torsades de pointes (TdP) on telemetry. In high-risk individuals, medications with reduced effects on cardiac conduction should be considered to avoid potentially lethal reactions. This case highlights the importance of clinical history prior to the administration of medications that have a propensity to cause abnormalities in cardiac conduction. Our patient had a grossly normal QT interval prior to the administration of azithromycin; however, she subsequently developed torsades de pointes. The patient was on telemetry monitoring, and cardiopulmonary resuscitation was quickly initiated as she was in a hospitalized setting; however, in an outpatient community setting, she likely would not have survived. By examining all the elements which contribute to QT prolongation, clinicians can have a deeper understanding of the complexities, particularly in individuals with multiple co-morbid conditions prior to the administration of medications that have a propensity to affect the QT interval.

## Introduction

Torsades de pointes (TdP) is defined as a polymorphic ventricular tachycardia characterized by a pattern of twisting of the points, which occurs when there is dysfunction in the repolarization of cardiac myocytes due to either a congenital or acquired prolonged QT interval [1-5]. QT prolongation with subsequent ventricular fibrillation was initially reported in response to quinidine in 1964 by Slezer and Wray [3]. In 1966 TdP was first depicted by Dessertenne as a polymorphic ventricular tachycardia in which the QRS complexes twisted around the isoelectric line in a sinusoidal fashion [4]. Drug-induced TdP resulting in cardiac arrest is a rare event; however, it is potentially disastrous even in hospitalized settings due to the propensity of transitioning into ventricular fibrillation [1-2].

## Case presentation

History of presentation

**Figure 1:**
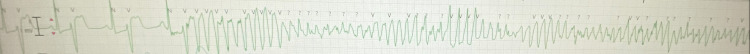
Torsades de pointes telemetry strip from cardiac arrest

Our case is a 71-year-old female visiting from Colombia who presented to the emergency room with a productive cough, subjective fever, and chills for the past three days. On initial presentation, she was alert and oriented, saturating 96% on room air. Other vital signs were blood pressure of 96/57, tachycardic to 114, respiratory rate of 18, and a temperature of 100.1 F. Home medications were aspirin, clopidogrel, dapagliflozin, sacubitril-valsartan, rosuvastatin, levothyroxine, nebivolol, omeprazole, and metformin. She admitted to recent contact with a relative with similar symptoms. She experienced episodes of hypotension to 80s systolic over 40s diastolic while in the emergency department and was evaluated by the medical intensive care unit. She was found to have fluid-responsive hypotension with point-of-care ultrasound demonstrating inferior vena cava contraction, indicative of dehydration. She received azithromycin and ceftriaxone intravenously as a treatment for possible pneumonia due to her symptoms and elevated procalcitonin of 0.63. She became unresponsive an hour after the completion of the intravenous antibiotic and had torsades de pointes (TdP) on telemetry (Figure 1). She developed cardiac arrest, and advanced cardiovascular life support was initiated. Return of spontaneous circulation was achieved after one minute of cardiopulmonary resuscitation. She was intubated for airway protection and placed in the intensive care unit.

Medical history

She has a history of hypertension, hyperlipidemia, type II diabetes mellitus, hypothyroidism, and coronary artery disease, with coronary artery bypass grafting in July 2022 in Colombia.

Differential diagnosis

Differential diagnoses for TdP included azithromycin, electrolyte abnormalities, hypothyroidism, genetic abnormalities, and cardiac ischemia.

Investigations

**Figure 2:**
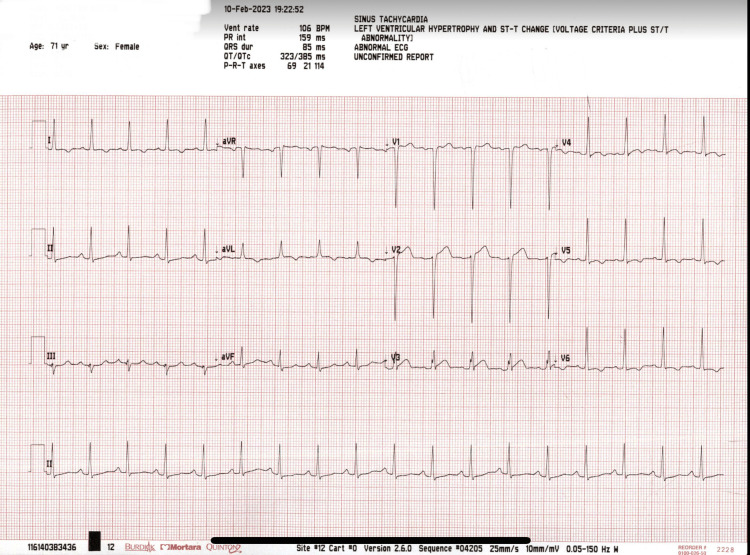
Initial electrocardiogram showing sinus tachycardia with left ventricular hypertrophy and normal QT interval

Laboratory investigations were significant for anemia with hemoglobin of 10.7, hyponatremia of 131 mmol/L, potassium of 3.9 mmol/L, magnesium of 1.80 mg/dL, pro-BNP of 3,049 pg/mL, negative troponin (<0.010 ng/mL) and lactate of 1.8 mmol/L. All other laboratory values were within normal limits. Urinalysis was negative for leukocyte esterase, nitrites, and bacteria. Urine and blood cultures were negative. Chest X-ray and bedside point-of-care ultrasound did not reveal any acute findings. Electrocardiogram demonstrated a QT interval of 385 milliseconds with left ventricular hypertrophy based on Cornell criteria and T wave inversions in leads V4, V5, and V6 (Figure 2).

Management

**Figure 3:**
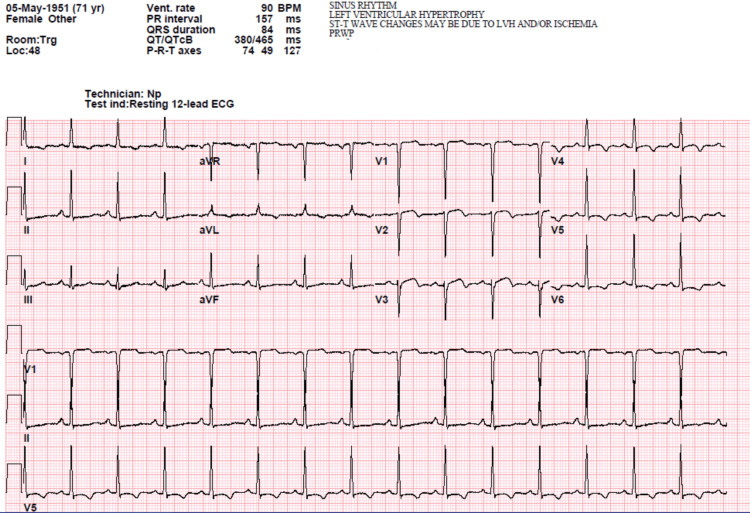
Post-cardiac arrest EKG demonstrated a prolonged QTc interval

Cardiology evaluation following the cardiac arrest recommended cessation of all QT-prolonging medications, including azithromycin, and administering magnesium sulfate 2 grams intravenously. Subsequently, her free T4 level was noted to be normal at 1.9 ng/dL. Thyroid-stimulating hormone (TSH) was severely depressed at 0.04 uIU/mL, indicating subclinical hyperthyroidism. Echocardiogram revealed a dilated left ventricle with hypokinesis of the inferior wall and interventricular septum, besides a large apical wall akinetic segment. Post-cardiac arrest EKG demonstrated a prolonged QTc interval of 465 ms (Figure 3). 

Follow-up

The patient underwent cardiac catheterization and was found to have triple vessel disease in her distal left anterior descending artery with 90% occlusion, mid-right coronary artery with 95% occlusion, and distal right coronary artery with 80% occlusion. The graft to the right coronary artery could not be viewed; however, a percutaneous coronary intervention was performed to the distal left anterior descending artery. A LifeVest was placed pending the placement of an implantable cardiac defibrillator (ICD). She was subsequently discharged home with outpatient follow-up.

## Discussion

Torsades de pointes (TdP) is defined as a polymorphic ventricular tachycardia characterized by a pattern of twisting of the points, which occurs when there is dysfunction in the repolarization of cardiac myocytes due to a prolonged QT interval, congenital or acquired [1-5]. QT prolongation with subsequent ventricular fibrillation was initially reported in response to quinidine in 1964 by Slezer and Wray [3]. TdP was first depicted in 1966 by Dessertenne as a polymorphic ventricular tachycardia in which the QRS complexes twisted around the isoelectric line in a sinusoidal fashion [4]. Though drug-induced TdP resulting in cardiac arrest is rare, it is potentially disastrous even in hospital settings due to the propensity of transitioning into ventricular fibrillation [1-2]. In the inpatient setting, clinicians must keep in mind that administration of medications with a propensity to prolong the QT interval is more likely to precipitate TdP compared to administration of the medication in the outpatient setting since hospitalized patients often have multiple risk factors which can provoke a new arrhythmia [1-5]. Although the prevalence of TdP is unknown in the 400,000 sudden cardiac deaths in the United States per year, torsades is estimated to account for less than five percent [5].

A reason for the proarrhythmic state of many hospitalized patients is that patients are often older, with underlying cardiac, renal, or hepatic dysfunction, and are receiving medications intravenously, resulting in a rapid response from the heart [1-5]. Prior to initiating therapies with a propensity to prolong the QT interval, it is crucial to obtain a baseline electrocardiogram to evaluate the QT interval.

Congenital QT prolongation occurs when at baseline, the QT interval is prolonged due to genetic mutations, resulting in alterations in the electrical conduction of the myocardium. Acquired QT prolongation occurs when the QT interval is lengthened due to precipitating factors such as electrolyte abnormalities, medications, or bradycardia. The three most common genes accounting for 80% of congenital prolonged QT cases are KCNQ1, KCNH2, and SCN5A [6]. One study compared acquired QT prolongation patients with congenital QT prolongation patients and demonstrated that one-third of patients with acquired prolonged QT were carriers of gene mutations responsible for congenital prolonged QT, such as the KCNH2 gene. The question arises whether genetic testing to identify carriers of QT-prolonging genes is a worthwhile, cost-effective venture, particularly in patients with significant comorbidity conditions [6].

Our patient presented with a normal QT interval, which makes one wonder whether inducible QT prolongation is secondary to severe coronary artery disease with subsequent myocardial damage, resulting in conduction abnormalities and, ultimately, arrhythmias. Polymorphic ventricular tachycardia with a normal QT interval has been reported as being associated with myocardial ischemia; however, this typically occurs in the hyperacute period of myocardial infarction due to ventricular extrasystoles with a very short coupling interval, resulting in R on T phenomenon. During the healing phase of myocardial infarction, polymorphic ventricular tachycardia has also been depicted in which patients have no evidence of acute ischemia but have a new prolongation of the QT interval, resulting in a pause-dependent infarct-related TdP [7]. Our patient did not demonstrate the characteristics of an acute myocardial infarction immediately preceding or following her developing TdP, and the QTc interval was prolonged at 465 ms after the return of spontaneous circulation (ROSC) was achieved. Polymorphic ventricular tachycardia with a borderline normal QTc interval has also been reported in the setting of electrolyte abnormalities such as hypokalemia and ryanodine receptor mutations of the myocardial and sodium channels [7]. While electrolyte abnormalities were not a factor in our patient, it is not known whether any genetic mutations were present which could have contributed to her developing TdP.

Another crucial factor in the case comprises the other non-cardiac comorbid conditions our patient had, such as hypothyroidism. Although hypothyroidism is a recognized but rare cause of TdP due to bradycardia and subsequent QTc prolongation, there have been few reports regarding the effects of hyperthyroidism on the QTc interval. One study compared the QTc intervals of 38 individuals with hyperthyroidism with age-matched control subjects without hyperthyroidism. The study found a positive correlation between higher free T4 levels and QTc intervals being prolonged or borderline. The study, however, did not find a correlation between TSH levels and QTc interval [8]. While our patient had a free T4 level within the reference range, she had a severely suppressed TSH, which leads one to question whether further studies are warranted to truly understand the effects of hyperthyroidism on cardiac functioning.

The central focus, particularly in this case, is the propensity of medications to induce QT prolongation. Medications can cause alterations in the sodium, calcium, or potassium channels of ventricular myocytes, resulting in prolonged action potentials and subsequent QT prolongation. Increases in inward currents via calcium or sodium channels or a reduction in outward currents via potassium channels result in QT prolongation by prolonging the ventricular myocytes' action potentials. Of these three mechanisms, medications responsible for acquired prolonged QT syndrome typically act on the delayed rectifier potassium current Ikr (rapid), which is one of the two potassium channels responsible for ventricular repolarization [9,10]. The IKr channel is encoded by the KCNH2 gene and there, it is theorized that patients who are carriers of KCNH2 mutations may have a predisposition to medication-induced QT prolongation [9-12]. Medications associated with QT prolongation have been theorized to disturb the migration of newly formed IKr proteins from the endoplasmic reticulum to the cell membrane, resulting in reduced IKr channel expression. Another theory regarding how certain medications promote TdP is the potentially increased expression of late sodium current INa-L channels [10]. While the mechanism of many QT-prolonging medications is not fully understood, certain medications, such as macrolides, have a well-established mechanism, besides a strong clinical correlation, to a higher incidence of ventricular arrhythmias [10-13]. Macrolides inhibit the rapidly activating IKr component while not acting on the slowly activating IKs component of the delayed rectifier IK current, resulting in prolonged repolarization, prolonged action potential duration, and subsequently, prolongation of the QT interval [11-13]. One study analyzed the incidence of ventricular arrhythmias or sudden death in patients who had not received macrolide therapy versus individuals who had received macrolides. In individuals not receiving macrolide antibiotics, there was an average of 80 cases of ventricular arrhythmias or sudden death per million patients treated. In patients receiving macrolide therapy, there were 118 cases of ventricular arrhythmias or sudden death per million treatments [10,12]. One study examining individuals with ischemic and dilated cardiomyopathy suggests that examining the beat-to-beat variability of the repolarization phase of the QT interval can be done to predict the development of ventricular arrhythmias. The study found that patients with ischemic cardiomyopathy tended to have an increased incidence of non-sustained ventricular tachycardia compared to those with dilated cardiomyopathy; however, due to small sample sizes, the difference lacked statistical significance. Further studies are needed to determine whether early investigations such as Holter monitoring for beat variability or interventions such as prophylactic ICD placement can aid in preventing mortality in these high-risk populations [14].

## Conclusions

Our case highlights the importance of careful consideration prior to the initiation of QT-prolonging agents in high-risk populations. In patients with a history of cardiomyopathy or severe coronary artery disease without underlying prolonged QTc on initial electrocardiogram, the risks and benefits must be carefully considered before initiating QT-prolonging agents, as these patients are at a much higher risk of a lethal arrhythmia, compared to the general population. In high-risk populations, alternative agents with a reduced propensity to affect the cardiac conduction system should be considered to avoid potentially lethal adverse events.
